# *APOBEC3B* gene overexpression in non-small-cell lung cancer

**DOI:** 10.3892/br.2014.256

**Published:** 2014-03-18

**Authors:** HIDEFUMI SASAKI, AYUMI SUZUKI, TSUTOMU TATEMATSU, MASAYUKI SHITARA, YU HIKOSAKA, KATSUHIRO OKUDA, SATORU MORIYAMA, MOTOKI YANO, YOSHITAKA FUJII

**Affiliations:** Department of Oncology, Immunology and Surgery, Nagoya City University Graduate School of Medical Sciences, Nagoya, Aichi 467-8601, Japan

**Keywords:** *APOBEC3B*, mutation, lung cancer, epidermal growth factor receptor, LightCycler

## Abstract

Recent study results have demonstrated that a subclass of apolipoprotein B mRNA-editing enzyme, catalytic polypeptide-like (APOBEC) cytidine deaminase may induce mutation clusters in various types of cancer. From the Cancer Genome Altas, an APOBEC mutation pattern was identified in bladder, cervical, breast, head and neck and lung cancers. In the present study, *APOBEC3B* mRNA expression was investigated using quantitative reverse transcription-polymerase chain reaction (RT-qPCR) assay using LightCycler in surgically treated non-small-cell lung cancer (NSCLC) cases. Additionally, 88 surgically removed Japanese NSCLC cases were analyzed for mRNA level. The results showed that *APOBEC3B/β-actin* mRNA levels were significantly higher in lung cancer (1,598.481±6,465.781) when compared to adjacent normal lung tissues (2,116.639±8,337.331, P=0.5453). The tumor/normal (T/N) ratio of *APOBEC3B/β-actin* mRNA levels was not different within the gender, age, smoking status and pathological stages. The T/N ratio of *APOBEC3B/β-actin* mRNA levels was not significantly different in epidermal growth factor receptor (*EGFR*) or *Kras* mutation-positive cases as compared to the wild-type cases.

## Introduction

Lung cancer is a leading cause of mortality due to its high incidence, malignant behavior and lack of major advancements in treatment strategy (1). Lung cancer was the leading indication for respiratory surgery (47.5%) in 2009 in Japan (2) and >30,000 patients underwent surgery for lung cancer at Japanese institutions in the same year (2). The clinical behavior of non-small-cell lung cancer (NSCLC) is largely associated with its stage. Treatment by surgery is only achieved in cases representing an early stage of NSCLC (3).

Genome instability triggers the development of many types of cancer (4,5). Normal enzymatic activities may be one of the sources of DNA damage and mutation. Cytidine deaminases, which convert cytosine bases to uracil, may contribute to DNA damage (6). The human genome encodes several homologous apolipoprotein B mRNA-editing enzyme, catalytic polypeptide-like (APOBEC) cytidine deaminases that function in innate immunity as well as in RNA editing (7). Recently, clustered mutations (termed kataegis) (8) identified through next-generation sequencing suggested that APOBECs may induce base substitutions in tumor genomes (8,9). Analysis of data pertaining to breast (10) and lung (11) cancer sequencing and expression suggested that it is specifically APOBEC3B that causes mutations in these types of cancer. Results of previous studies have shown that other members of the APOBEC family occasionally gain access to the nucleus and cause cancer-associated genomin damage or mutation (12–14). Despite the indication that APOBEC-mediated mutagenesis plays a role in cancer, it was unclear how strong of a mutagenic factor APOBEC enzymes are, whether APOBEC mutagenesis is a ubiquitous characteristic of many cancer types, and cases and whether it is associated with any specific tumor characteristics or development.

In this study, *APOBEC3B* mRNA expression was investigated in Japanese NSCLC and adjacent normal lung tissues using quantitative polymerase chain reaction (qPCR) using LightCycler (Roche Molecular Biochemicals, Mannheim, Germany) in surgically treated cases. The findings were compared to the clinicopathologic characteristics of NSCLC and *APOBEC3B* gene status.

## Patients and methods

### Patient samples

The study group included NSCLC patients who had undergone surgery at the Department of Surgery, Nagoya City University Hospital between 2006 and 2010. Tumor samples were immediately frozen and stored at −80°C until they were assayed. The clinical and pathological characteristics of the 88 NSCLC patients for *APOBEC3B* mRNA gene analyses were as follows: 59 (67.0%) were male and 29 (33.0%) were female; 65 (73.9%) were diagnosed as adenocarcinomas and 21 (23.9%) were squamous cell carcinoma; 58 (65.9%) were smoker and 30 were non-smoker; 59 (67.0%) were pathological stage I.

### PCR assay for APOBEC3B gene

Total RNA was extracted from NSCLC and adjacent normal lung tissues using an Isogen kit (Nippon Gene, Tokyo, Japan) according to the manufacturer’s instructions. RNA concentration was determined by a NanoDrop ND-1000 spectrophotometer (NanoDrop Technologies, Inc., Rockland, DE, USA). Approximately 10 cases were excluded for each assay as tumor or normal cells were not sufficient for tumor extraction or tumor RNA. RNA (1 μg) was reverse transcribed by First Strand cDNA Synthesis kit with 0.5 μg oligo (dT)_16_ (Roche Diagnostics GmbH, Mannheim, Germany) according to the manufacturer’s instructions. The reaction mixture was incubated at 25°C for 15 min, 42°C for 60 min, 99°C for 5 min and then at 4°C for 5 min. The cDNA concentration was determined by NanoDrop ND-1000 spectrophotometer (NanoDrop Technologies, Inc., Rockland, DE, USA). Approximately 200 ng of each cDNA was used for PCR analysis. To ensure the accuracy of mRNA extraction and reverse transcription, the samples were subjected to qPCR amplification with a *β-actin* primers kit (Nihon Gene Laboratory, Miyagi, Japan) using a LightCycler FastStart DNA Master HybProbe kit (Roche Diagnostics GmbH). The *APOBEC3B* qPCR assay reactions were performed using LightCycler FastStart DNA Master SYBR-Green I kit (Roche Diagnostics GmbH) in a 20-μl reaction volume. The primer sequences used for the *APOBEC3B* gene were: forward, 5-TCCTTCAGGAGAACACACAC-3 and the reverse, 5-TCGTAGGTCATGATGGAGAC-3 (130 bp). The cycling conditions used were: initial denaturation at 95°C for 10 min, followed by 40 cycles at 95°C for 10 sec, 56°C for 10 sec and 72°C for 6 sec.

### Statistical analysis

Statistical analysis was performed using the Student’s t-test for unpaired samples and Wilcoxon’s signed-rank test for paired samples. Correlation coefficients were determined by rank correlation using Spearman’s test. Overall survival of lung cancer patients was assessed by the Kaplan-Meier methods and differences were examined by the log-rank test. Analysis was performed using the Stat-View software package (Abacus Concepts, Inc., Berkeley, CA, USA) and was considered significant when P<0.05.

## Results

### APOBEC3B mRNA status in Japanese lung cancer patients

We quantified the *APOBEC3B* gene status for 88 NSCLC samples and adjacent normal lung tissues. The *APOBEC3B/β-actin* mRNA levels were significantly higher in the lung cancer (24.780±42.625) when compared to the adjacent normal lung (4.176±7.770) tissues (P<0.0001). Tumor/normal (T/N) ratio of the *APOBEC3B/β-actin* mRNA level did not correlate with gender (male vs. female, P=0.4694), ages (age ≤65 vs. >65, P=0.9050) and smoking status (smoker vs. non-smoker, P=0.1151). The T/N ratio of the *APOBEC3B*/*β-actin* mRNA level did not correlate with pathological stages and subtypes (adenocarcinoma vs. others, P=0.5031). The T/N ratio of the *APOBEC3B/β-actin* mRNA level was not significantly different in lymph node metastasis (3.871±5.546) and the lymph node metastasis-negative cases (4.278±8.416, P=0.9462) (Table I). The T/N ratio of *APOBEC3B/β-actin* mRNA level was not significantly different in *Kras* mutation-positive (8.36±16.869) and the *Kras* mutation-negative (3.87±6.774) cases (P=0.8361). The T/N ratio of *APOBEC3B/β-actin* mRNA level was not significantly different in epidermal growth factor receptor *(EGFR)* mutation-positive (6.849±8.882) and the *EGFR* mutation-negative (3.488±7.341) cases (P=0.1534) (Table I).

## Discussion

In this study, we have shown that *APOBEC3B* mRNA expression was significantly higher in NSCLC than in adjacent normal lung. No correlation was found between *APOBEC3B* expression and clinicophatological characteristics, such as stage and pathological subtypes indicating that *APOBEC3B* gene may be involved in the tumorigenesis of NSCLC. The major mutations, *EGFR* and *Kras*, were not converted from cytosine bases to uracil. Thus, no correlation between *APOBEC3B* expression and these mutations was found.

Sequencing of cancer genomes has revealed that tumors contain numerous mutations (15). It is unclear how such a large number of DNA sequence changes are induced and what factors influence the distributions of the rates of mutations among cells and within regions of the genome during tumor development. Whole genome sequencing of 21 breast cancers recently revealed the presence in more than half the cancers of a novel form of localized hypermutation (8). APOBEC cytidine-DNA deaminases may therefore be involved in the process (8,16). Members of the APOBEC family enzymes, of which there are seven in humans, deaminate cytosine in the context of a single-strand polynucleotide substrate and have a function in adaptive and innate immunity.

APOBEC3 family members act on C residues in the DNA of viral replication intermediates as part of a host restriction pathway. Expression of APOBEC cystidine deaminases in yeast generates mutations across the genome, a portion of which are found in clusters (17). Cystidine deaminases have been shown to generate such clustered mutations (18,19). In the present study, we examined whether *APOBEC3B* may also act as a molecule for tumor progression and metastasis in NSCLCs. In our analyses, *APOBEC3B* was highly expressed and common in NSCLC tumor cells. However, *APOBEC3B* expression did not correlate with metastasis or tumor invasion.

The mutation data obtained in yeast reveal APOBEC3B and APOBEC3A as the only deaminases characterized whose target specificity matches the breast cancer kataegic mutations, strongly suggesting involvement of these deaminases in cancer kataegis (17). Burns *et al* (10) have demonstrated that APOBEC3B expression correlated with a T-C mutator phenotype in many primary breast cancer tumors. In addition, APOBEC3B was well expressed in NSCLC samples (10). However, APOBEC3A also incuded mutation of human papilloma DNA (14) and transfected plasmid DNA (20). Enforced expression of APOBEC3A has also shown to lead genomic damage in the nucleus (21). It remains possible that other APOBEC3s may contribute to genome mutation in other tumors.

Determining the mutagenic factors that underlie the mix of mutations in tumors is important for a general understanding of carcinogenesis. Previously, it was shown that the APOBEC mutagenesis pattern induced cancer driver genes (11). Despite a positive correlation between APOBEC3B expression and APOBEC-mediated mutagenesis, the extent of the association was relatively small (Spearman’s r=0.30). These factors presumably contribute to APOBEC-mediated mutagenesis.

## Figures and Tables

**Table I:** Clinicopathological characteristics of 88 lung cancer patients.

	*APOBEC3B*		
	
Factors	No. of patients (%)	T/N ratio of *APOBEC3B/β-actin* mRNA levels	P-value
Mean age (years) 66.9±9.2	88		
Stage			
I	59 (67.0)	4.640±8.820	NS
II	14 (15.9)	2.830±3.77	
III–IV	15 (17.0)	3.606±6.050	
Tumor status			
T1	37 (42.0)	3.095±6.140	NS
T2	37 (42.0)	6.099±9.947	
T3	5 (5.7)	3.462±3.710	
T4	9 (10.2)	1.110±1.170	
Lymph node metastasis			
N0	66 (75.0)	3.871±5.546	0.9462
N1–2	22 (25.0)	4.278±8.416	
Age (years)			
≤65	35 (39.8)	3.351±4.830	0.4214
>65	53 (60.2)	4.721±9.210	
*EGFR* mutation			
Positive	18 (20.5)	6.849±8.882	0.1534
Negative	70 (79.5)	3.488±7.371	
Smoking			
BI=0	30 (34.1)	4.240±6.158	0.1151
BI>0	58 (65.9)	4.140±8.535	
Pathological subtypes			
Adeno	65 (73.9)	4.676±8.551	0.5031
Non-adeno	23 (26.1)	2.746±4.814	
Gender			
Male	59 (67.0)	3.456±6.674	0.4694
Female	29 (33.0)	5.641±9.592	

*APOBEC3B*, polipoprotein B mRNA-editing enzyme, catalytic polypeptide-like; T/N, tumor/normal; NS, not significant; *EGFR*, epidermal growth factor receptor; BI, Brinkman index.
